# Physical and Mechanical Properties of Particleboard Produced with Addition of Walnut (*Juglans regia* L.) Wood Residues

**DOI:** 10.3390/ma15041280

**Published:** 2022-02-09

**Authors:** Marta Pędzik, Radosław Auriga, Lubos Kristak, Petar Antov, Tomasz Rogoziński

**Affiliations:** 1 Wood Technology Centre, Łukasiewicz Research Network, Poznań Institute of Technology, 60-654 Poznań, Poland; marta.pedzik@pit.lukasiewicz.gov.pl; 2 Faculty of Forestry and Wood Technology, Poznań University of Life Sciences, 60-627 Poznań, Poland; 3 Institute of Wood Sciences and Furniture, Warsaw University of Life Sciences—SGGW, 02-787 Warsaw, Poland; radoslaw_auriga@sggw.edu.pl; 4 Faculty of Wood Sciences and Technology, Technical University in Zvolen, 960 01 Zvolen, Slovakia; kristak@tuzvo.sk; 5 Faculty of Forest Industry, University of Forestry, 1797 Sofia, Bulgaria; p.antov@ltu.bg

**Keywords:** alternative raw material, walnut, applied pressure, wood residues, resin content, particleboard, physical and mechanical properties

## Abstract

The depletion of natural resources and increased demand for wood and wood-based materials have directed researchers and the industry towards alternative raw materials for composite manufacturing, such as agricultural waste and wood residues as substitutes of traditional wood. The potential of reusing walnut (*Juglans regia* L.) wood residues as an alternative raw material in particleboard manufacturing is investigated in this work. Three-layer particleboard was manufactured in the laboratory with a thickness of 16 mm, target density of 650 kg∙m^−3^ and three different levels (0%, 25% and 50%) of walnut wood particles, bonded with urea-formaldehyde (UF) resin. The physical properties (thickness swelling after 24 h) and mechanical properties (bending strength, modulus of elasticity and internal bond strength) were evaluated in accordance with the European standards. The effect of UF resin content and nominal applied pressure on the properties of the particleboard was also investigated. Markedly, the laboratory panels, manufactured with 50% walnut wood residues, exhibited flexural properties and internal bond strength, fulfilling the European standard requirements to particleboards used in load-bearing applications. However, none of the boards met the technical standard requirements for thickness swelling (24 h). Conclusively, walnut wood residues as a waste or by-product of the wood-processing industry can be efficiently utilized in the production of particleboard in terms of enhancing its mechanical properties.

## 1. Introduction

The global demand for wood and wood-based materials is constantly increasing. Using wood more efficiently to meet projected demands for the production of wood-based panels is a key circular economy principle [1,2]. The growing environmental concerns and recent legislative regulations, related to promoting the cascading use of natural resources, have posed new challenges to both the wood-based panel industry, related to the optimization of the available wood and other lignocellulosic raw materials, recycling, reusing wood and wood-based composites, and the search for alternative resources [3,4,5].

A problem for many companies and producers of wood and wood-based products is the insufficient amount of wood on the local market, which results in significant competition between wood-based industries. This competition will become more and more intense due to the expanding production capacities resulting in greater supply as a response to the growing demand [6,7]. The factors affecting the timber market and the increase in the price of timber are random and can occur at any time. The increase in wood prices may be caused by the global economic crisis, i.e., market and economic conditions. In turn, the fall in wood prices is often caused by natural disasters and factors, such as storms, e.g., in Italy or Austria [8]. The occurrence of bark beetle in spruce in the Czech Republic contributed to the deterioration of the quality of the raw material and the need for unplanned logging, and almost 100 million m^3^ of wood was obtained, which resulted in losses of approximately EUR 1.12 billion in the forestry sector [9]. In 2020, the prices of industrial timber logs delivered in the United States increased by about 2.5% for all classes and species combined compared to the previous year [10]. The average selling price of sawmill softwood in September 2021 in the United Kingdom was around GBP 79 per cubic meter of bark in nominal terms, and a year earlier the price was around GBP 50 [11]. There is also an increase in the average price of round wood obtained, e.g., by the Polish forest inspectorates by 7.8% in 2020 compared to the previous year, and by 11.1% compared to 2016 [12,13,14]. The export of significant amounts of unprocessed wood is another reason for the limited availability of wood raw material. The import of wood from other countries is associated with additional transport costs and emissions of harmful compounds. In Europe, the emphasis is on pro-ecological activities and reducing CO_2_ emissions. However, since 2015, the amount of exported industrial roundwood from European countries has increased from approx. 66 million m^3^ to almost 78.5 million m^3^ in 2020 [15].

The wood-based panel industry has certain flexibility about the use of raw materials, caused by the continuously changing wood raw material situation or regional variations of wood supplies. Moreover, the increased demand from other wood-based industries and the energy sector for wood previously used mainly for wood-based panel manufacturing has significantly increased worldwide [16]. These challenges have forced the wood-based sector to shift towards alternative raw materials, including recovered wood and by-products from other forest-based industries, as well as to optimize the technological production processes in order to maintain a consistent quality level.

Particleboards are one of the most important value-added panel products in the wood-based industry with a wide variety of applications [17,18]. Compared to the pulp and paper industry or construction, the production of particleboards can utilize low-quality raw materials. Proper waste management, including wood and wood-based by-products, is of great importance for the environment [19]. Many authors have investigated the potential applications of a particular material or the selection of appropriate manufacturing conditions, such as the type and amount of adhesive used or the temperature and pressure applied [20,21,22,23,24,25,26,27]. In the case of the expected deterioration of the technological properties of the boards with the addition of various alternative lignocellulosic raw materials, one possible way to counteract these undesirable effects is to increase the amount of binder [28]. The selection of the resin type and content is made on the basis of assumptions regarding the selected properties and projected applications. If the board is to have high water resistance, choose a resin other than UF or modify it with a different resin, e.g., PF (phenol-formaldehyde) or pMDI (polymeric 4,4′-methylenediphenyl isocyanate) [29,30,31,32,33,34,35]. This is a common procedure when using particles, e.g., annual plants, which will ensure good bonding and strength parameters [20,36]. The use of alternative raw materials contributes to the sustainable management of unused forest biomass, including bark, harvested and production residues, such as unprocessed sawmill by-products, i.e., less valuable wood waste from processing wood, such as sawdust, wood chips, shavings and wood pulp, and by-products of the food and agricultural industries, but also results in decreased panel production costs [3,37,38,39,40]. The increased shortage of wood raw materials, i.e., full-value wood and roundwood, justifies the wider industrial utilization of wood residues and by-products for wood-based panel manufacturing.

Particleboards can be fabricated from crushed lignocellulosic particles of one or more substitute raw materials, including post-consumer wood and wood from fruit trees and urban greenery [3,41,42,43,44]. Recycling of waste from construction and demolition in the form of residual medium density fiberboard (MDF), particleboard, cardboard and plywood is a viable option for producing boards suitable for furniture and interior applications [5,45]. Recently, many studies have been focused on the production of particleboard from alternative lignocellulosic raw materials, such as vine stalks [46], cotton stalks [26], bamboo and banana chips [47,48], poppy husks [49], wheat and straw [25,50], seaweed [51] and even chicken feathers [52,53]. Boards made with the addition of these materials should have comparable properties to industrially produced boards from softwood particles and comply with the requirements of the technical standard EN 312 about the specification of particleboards [54].

One of the possibilities of using alternative wood resources as a feedstock for particleboard production is the raw material created during care or liquidation of plantations as well as the cutting of old walnut trees. The total global area harvested of walnut plantations in 2019 amounted to approx. 9.3 million ha, including approx. 143,507 ha in Europe and 2270 ha in Poland [55]. These trees are cultivated worldwide not only for obtaining their edible nuts, but also for the production of decorative veneers, which is a very exacting and expensive process [56]. In turn, the price of wood residues suitable for particleboard is much lower. Due to the high price and low availability of walnut wood on the market, its use in particleboard production is feasible only in the case of wood residues and by-products from the wood-processing industry as a sustainable solution to the increased global demand for raw material.

The aim of this research work was to investigate the effect of the content of walnut (*Juglans regia* L.) wood residues on the physical and mechanical properties of three-layer particleboard as a way to alleviate the shortage of raw materials in the wood-based panel industry. The effect of reduced urea-formaldehyde (UF) resin content and applied pressure on the exploitation properties of the particleboard was also evaluated. This paper is a continuation of the research carried out by the authors on the possibility of using alternative raw materials for the production of particleboard.

## 2. Materials and Methods

### 2.1. Materials

Industrially produced softwood particles were obtained from the local particleboard factory. The walnut wood shavings were made from a medium-sized walnut tree stem obtained from a backyard cut in Dreglin, Poland. The harvested wood was debarked and then shredded into chips with a knife chipper. The produced chips were ground on a Pallmann laboratory cutter (Pallmann GmbH, Zweibrücken, Germany) in the form of particles and dried to a moisture content of approx. 8%, which was determined by the drying-weighing method according to the EN 322 standard [57]. The material was then sorted using screens with a mesh diameter of 4 mm and 2 mm, in order to select the material for the surface layers and the core layer. The desired fraction for the core layer consisted of particles retained on a sieve with a mesh size of 2 mm. Particles larger than 4 mm were reground and sorted, and particles smaller than 2 mm were used for the surface layers. The reason for the selection of the indicated sizes of vortices used in individual layers was the use of chip mixtures dimensionally similar to the dimensions of industrial chips used in the production of particleboards.

### 2.2. Adhesives

Commercial urea-formaldehyde (UF) resin with a molar ratio of 1.2, supplied by the factory Silekol Sp, z o.o. (Kędzierzyn-Koźle, Poland), was used for the production of the particleboards. The selected properties of the resin are presented in Table 1. Ammonium sulfate ((NH_4_)_2_SO_4_) was used as a hardener at a 10% water solution, and mixed with the resin before spraying into the wood particles. The formulation of the adhesive was 50:15:1.5 parts by weight of the resin, water and hardener, respectively. The proportions were selected to obtain the appropriate gel time of the adhesive mass. In addition, 0.8 wt.% paraffin emulsion, based on the dry particle weight, was added to the resin in order to protect the produced boards against exposure to water and to maintain the dimensional stability.

### 2.3. Production of Panels

The particle mixes were glued with the UF resin at a 12% resin content of the surface layers (SL) and a 10% resin content of the core layer (CL), based on the mass of the oven dry wood particles, using pneumatic spraying. The differences in the resin content were due to the different sizes and surface area of the particles used for the individual layers. The mixture of wood particles and resin was manually formed into a mat in a frame with 320 mm × 320 mm dimensions and pressed using aluminum plates and spacer bars. Three-layer particleboard with a thickness of 16 mm, target density of 650 kg·m^−3^ and three different addition levels (0%, 25% and 50%) of walnut particles, bonded with UF resin, were produced under laboratory conditions. The share of surface layers in the panel was 35 wt.%. The hot-pressing process was carried out in a ZUP-NYSA PH-1LP25 single opening hydraulic laboratory press using standard particleboard manufacturing conditions, i.e., a pressing temperature of 180°C, unit pressure of 2.5 N·mm^−^^2^, and pressing time of 20 s·mm^−1^ of the board thickness.

The manufacturing parameters of the laboratory-fabricated particleboard are given in Table 2.

The manufactured boards were conditioned at a temperature of 20 ± 2 °C and a relative air humidity of 65 ± 5% for 7 days. The manufactured boards were cut into the required test size in accordance with the relevant standards and subjected to tests to evaluate their physical and mechanical properties. The bending strength (MOR) and modulus of elasticity (MOE) were determined according to the EN 310 standard; the internal bond (IB) was determined according to EN 319. The thickness swelling (TS) after 24 h of soaking in water was determined according to EN 317. Each property was determined on 10 replicates for a given variant of the boards. The significance of the differences between the values of the individual panel parameters was calculated using the Tukey’s post hoc HSD test. The experimental data was statistically analyzed using STATISTICA 13.3 software (TIBCO Software Inc., Palo Alto, CA, USA).

## 3. Results

**Figure 1:**
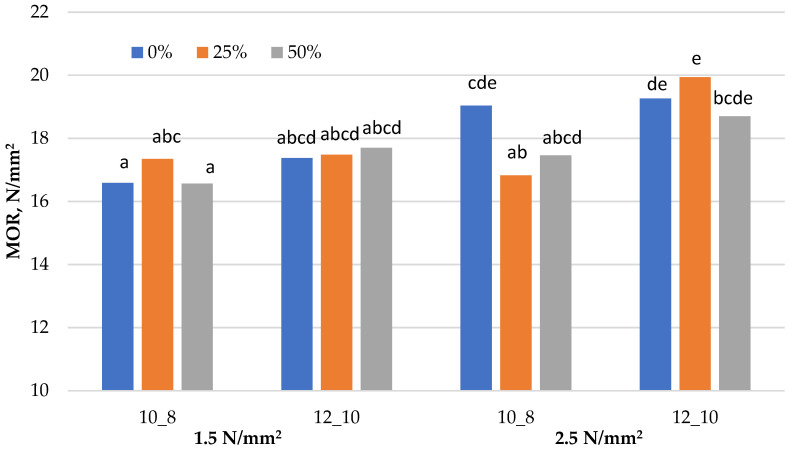
Bending strength (MOR) of particleboards produced.

**Figure 2:**
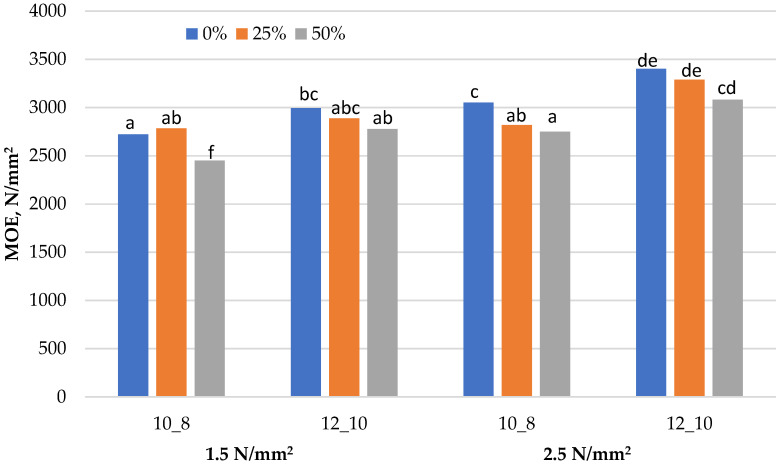
Modulus of elasticity (MOE) of particleboards produced.

**Figure 3:**
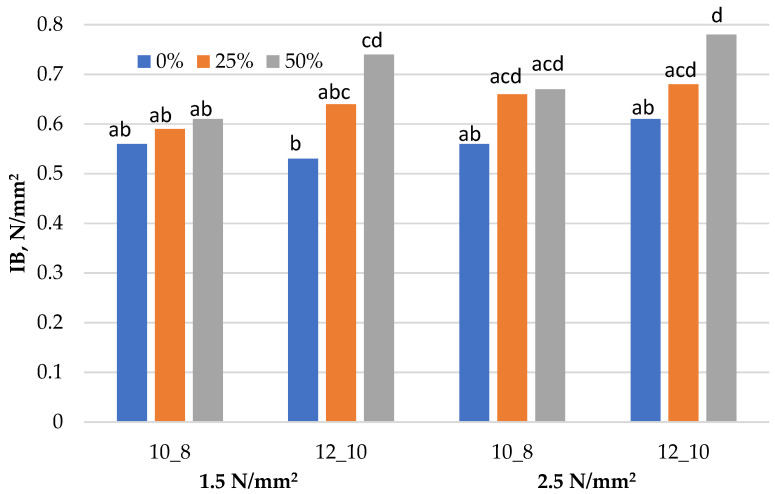
Internal bond (IB) of particleboards produced.

Graphical representation of the results obtained for the mechanical properties (MOR, MOE and IB) of the laboratory-produced three-layered particleboards, fabricated with a different share of residual walnut wood particles, is presented in Figure 1, Figure 2 and Figure 3. Table 3 presents the minimum requirements that the boards must meet in terms of mechanical and swelling properties in order to qualify them to the particular types, P5, P6 or P7. The results obtained for the TS (24 h) are presented in Table 4. All assumed variants of boards, both in terms of the level of substitution and technological factors, were successfully produced in accordance with the experimental design. Samples for testing were obtained from them and the determination of each property was made for at least 10 replicates for a given variant of the board.

### 3.1. Bending Strength (MOR)

The analysis of the obtained results demonstrates that the addition of walnut wood did not significantly affect the MOR value of the boards, even with the 50% addition of walnut particles (Figure 1). At low applied pressure, neither the walnut content nor the degree of sizing changed the MOR value. On the other hand, increasing the pressing pressure significantly improved the MOR for softwood boards. The increased pressure and resin content resulted in increased MOR values of the boards, fabricated with the addition of walnut wood particles. For these technological parameters, no statistical differences were found for boards with a 25% and 50% share of walnut wood.

The boards produced at a pressure of 1.5 N·mm^−^^2^ in the entire range of substitution demonstrated similar MOR values, which were also confirmed by the statistical analysis. These values were assigned to the homogeneous group marked with the letter a in Figure 1. Significant differences to the boards produced with a low applied pressure were observed for the MOR values of the boards produced with low resin content and higher applied pressure. The MOR value of particleboard fabricated of softwood particles was only approx. 19 N·mm^−^^2^ for both resin contents used at a pressure of 2.5 N·mm^−^^2^. Similar values were obtained for boards manufactured with walnut wood particles at the highest values of production parameters. The 25% and 50% share of walnut wood particles in the composition of laboratory-fabricated panels resulted in decreased MOR value of the boards bonded with resin content of 10% and 8%, and of the softwood particleboard by 11.6% and 8%, respectively, resulting in the achievement of parameters characteristic for the boards from the group with an applied pressure of 1.5 N·mm^−^^2^. These differences, although slight, were confirmed by the statistical analysis. As a result, eight variants of panels were classified into the homogeneous group marked with the letter a, and the remaining 4 were classified as group e.

Boards manufactured according to the standard conditions in the entire range of walnut wood particles substitution, and the board manufactured with softwood particles under standard pressure conditions, with a lower resin content, met the high requirements of technical standards for construction boards, which is type P6 (heavy-duty load-bearing boards for use in dry conditions). The remaining boards complied with the standard requirements for P5 type boards, i.e., load-bearing boards for use in humid conditions.

Different letters denote a significant difference. Means followed by the same letter do not statistically differ from each other (*p* ≤ 0.05) according to Tukey’s post hoc test.

### 3.2. Modulus of Elasticity (MOE)

In terms of MOE, all of the manufactured boards met the minimum standard requirements for P5, i.e., load-bearing boards for use in humid conditions—2400 N·mm^−2^. Boards made of softwood particles only demonstrated the highest MOE values for most variants in the range of 2721–3403 N·mm^−2^, as shown in Figure 2. The addition of residual walnut wood particles resulted in decreased MOE values in all variants produced. However, there was a slight decrease of up to 10% between the board without walnut wood and the board with its 50% substitution. However, in many cases, these changes were statistically insignificant. With the increase of both resin content and the applied pressure, the MOE value of the produced boards increased. More significant effect on the stiffness of the boards was observed for the applied pressure change. For the board fabricated with a 12% resin content of the SL and 10% of the CL, along with increasing the applied pressure, an increase in MOE values by 13.6%, 13.8% and 10.9%, about the respective share of residual walnut wood, was observed, respectively.

Different letters denote a significant difference. Means followed by the same letter do not statistically differ from each other (*p* ≤ 0.05), according to Tukey’s post hoc test.

### 3.3. Internal Bond (IB) Strength

Due to the relatively lower number of particles in the boards with increased walnut wood content, there is a better coating of each particle with the UF resin. This resulted in higher IB values, determined for the particleboard fabricated with the 50% share of walnut particles and higher, in particular, with an additionally increased degree of sizing. The analysis of the IB results obtained demonstrated that the substitution of softwood particles with walnut wood particles increased the tensile strength for each variant of the boards. The highest IB values in each variant of applied pressure and resin content were obtained for the particleboard manufactured with a 50% share of walnut wood, i.e., minimum 0.61 N·mm^−2^ and maximum 0.78 N·mm^−2^, and 0.59 N·mm^−2^ and 0.68 N·mm^−2^ for the boards fabricated with a 25% walnut wood share, respectively. The improvement of IB was particularly noticeable for boards bonded with the higher resin content, i.e., 12% and 10%. At a 50% share of walnut particles, the IB value was about 28% higher than the reference board produced at a pressure of 1.5 N·mm^−2^ and almost 40% higher than the same variants produced at the higher pressure.

All laboratory-produced particleboards fulfilled the requirements of technical standards for P6 boards, i.e., heavy-duty load-bearing boards for use in dry conditions, and the boards with maximum walnut particles substitution produced at a pressure of 2.5 N·mm^−2^ also met the most stringent standard minimum requirements for boards of type P7, i.e., heavy-duty load-bearing boards for use in humid conditions, exceeding it significantly.

Different letters denote a significant difference. Means followed by the same letter do not statistically differ from each other (*p* ≤ 0.05) according to Tukey’s post hoc test.

### 3.4. Thickness Swelling (TS)

The results of the TS (24 h) of the laboratory-produced three-layer particleboards are presented in Table 4.

At a lower degree of sizing of 50%, the addition of walnut wood deteriorated the TS properties due to the swelling of the particles, which have a higher density. By increasing the amount of resin, the absorption of water by the particles is made difficult, even if the resin used does not have high water resistance. With the standard resin content of 12% of the SL and 10% of the CL, this parameter remained at the level of 20.7–21.7% for the entire range of wood particle substitution and both variants of applied pressure.

These slight differences were statistically insignificant for all panels manufactured with this resin content. The reduction in the amount of the UF resin resulted in deteriorated dimensional stability of the boards. The TS value of boards produced with the resin content of 10% of the SL and 8% of the CL was significantly higher than the boards with the standard resin content. In addition, the 50% share with the residue walnut wood particles contributed the most to increasing the TS value of boards produced with the lower applied pressure. TS values of the boards produced with 50% share of walnut wood and bonded with a reduced resin content were 5% higher at 1.5 N·mm^−2^ and 10% higher at 1.5 N·mm^−2^, compared to the board fabricated from only pine particles. With regards to the board manufactured under standard conditions, the difference was 23% and 26%, respectively. None of the boards met the minimum requirements of technical standards in terms of TS (24 h) values, amounting to 14% for the P3 type board, i.e., non-load-bearing boards for use in humid conditions, and 10% for the P5 type board, i.e., load-bearing boards for use in humid conditions.

## 4. Discussion

Taking into account the variable content of walnut wood, two resin contents were selected in the study. This was to check whether this treatment would compensate for the decrease in mechanical properties associated with the use of wood other than pine. It turned out that the addition of walnut wood residues did not decrease the mechanical properties. In addition to the improved mechanical properties, the increased amount of UF resin improved the dimensional stability of the boards by reducing the water absorption of the lignocellulosic particles.

Urea-formaldehyde (UF) adhesives are the most widely used thermosetting resins for the production of various types of wood-based composites [58]. The wide industrial use of these resins is due to their good adhesion performance, high reactivity, water solubility, short press times and a relatively low price [17,59]. Due to the very variable and complex nature of the wood raw material, the properties of wood-based panels are largely determined by the characteristics of the resin [58].

The mechanical properties, e.g., MOE, MOR and IB values of boards manufactured with willow (*Salix viminalis* L.) wood for the core layer, improved with an increased resin content from 8% to 9.5% by 10% and 20%, respectively [27]. It was also found that increasing the resin content by up to 16% and the pressing pressure had a significant impact on the mechanical and physical properties of the particleboard fabricated from non-wood raw materials. This results in a better filling of the voids between the particles, improving the compaction of the panels, thus leading to the improvement of the mechanical and physical properties [60]. In addition, increasing the amount of resin from 8–10% to 10–12% may also lower the surface roughness values in boards with particles and dust wood [61].

However, a significant disadvantage of this commonly used thermosetting amino resin is the low water-resistance, which has been confirmed by tests. Low water resistance values were observed for all variants of the produced experimental boards, which mean that the manufactured products are intended to be used mainly in internal conditions. Increasing the gluing degree of the boards resulted in a decrease in TS value from 25.9% to 21.6% for a pine board and from 24.7% to 21.3% for a board with a 25% share of walnut. Additionally, increasing the pressure from 1.5 N·mm^−2^ to 2.5 N·mm^−2^ lowered the TS value to 21.7%, 20.7% and 21.5% for the board with 0%, 25% and 50% walnut content, respectively. The lowest TS value of 20.7% was determined for the boards manufactured with 25% share of walnut wood particles. The properties of particleboard can vary depending on the resin content. Perhaps, if an adhesive with greater resistance to water, e.g., phenol-formaldehyde resin, was used, the results would be much better.

For spruce, sunflower and topinambour (*Helianthus tuberosus*) particles, a slight improvement in TS was also obtained, by a maximum of 8% and approx. 2% for the remaining ones [62]. However, for pine and white mustard straw, increasing the amount of glue from 10% to 14% resulted in a reduction of TS from 51% to 22% and from 62% to 39%, respectively [28]. Nevertheless, better results for panels made of lignocellulosic particles, including annual plants and cereals, can be achieved by using adhesives more dedicated to such raw materials, i.e., pMDI [62,63].

## 5. Conclusions

The conducted research proved that it is possible to add walnut wood residues to commonly produced softwood particleboard. However, it should not be expected that there will be a lot of this wood, because it is a remnant, not a wholesome quantitatively and qualitatively assortment, similarly to recycled wood particles, residues from fruit orchards, urban wastes or residues from the production of wood products or food production waste.

The boards produced with higher resin content and higher applied pressure achieved the highest MOR values. For boards produced with a reduced applied pressure of 1.5 N·mm^−2^, no significant differences were found between the MOR values in terms of the share of residue walnut wood, even up to 50%, and a change in the resin content. The greater the addition of walnut wood particles, the more visible was the deterioration of MOE, and the greater the improvement of IB values. A higher proportion of the UF resin had a positive effect on the dimensional stability of the boards, while the effect of the addition of the walnut wood particles and the change in pressure were not statistically confirmed. Perhaps, if the resin used were modified to add water-resistant resins, the boards would meet the requirements of the standards throughout, also in terms of thickness swelling.

In terms of mechanical properties, all laboratory-produced boards exhibited very high MOR, MOE and IB values, and even the panels, fabricated with 50% walnut wood content, met the requirements of the technical standards for P5 boards, i.e., load-bearing boards for use in humid conditions, and in some cases, even in P6 and P7 variants, i.e., for load-bearing applications. It can be concluded that residual walnut wood particles can successfully replace softwood particles in the production of three-layer particleboard, which can be used in structural applications. In order to improve the resource efficiency and achieve enhanced valorization of waste biomass, future research should be aimed at the rational use of the available wood and lignocellulosic raw materials, the search for alternative resources and the optimization of production parameters.

## Figures and Tables

**Table 1:** Selected properties of UF resin used in this work.

Characteristic	Value
Dry solids	67%
Relative density	1.30 g∙cm^−3^
pH	8.0
Gel time	50 s
Dynamic viscosity	0.5 Pa∙s

**Table 2:** Manufacturing parameters of particleboard fabricated from industrial wood particles and residual walnut wood particles bonded with UF resin.

Share of Walnut Wood Particles [%]	UF Resin Content of the Surface Layers and Core Layer (SL_CL) [%]	Unit Pressure [N·mm^−2^]
0	10_8	1.5
25		
50		
0	12_10	
25		
50		
0	10_8	2.5
25		
50		
0	12_10	
25		
50		

**Table 3:** Requirements in terms of mechanical properties and swelling for particleboards.

* Property	Unit	Requirements for a Thickness Range > 13 to 20 mm		
		Type P5	Type P6	Type P7
Bending strength	N/mm^2^	16	18	20
Modulus of elasticity in bending	N/mm^2^	2400	3000	3100
Internal bond	N/mm^2^	0.45	0.50	0.70
Swelling in thickness, 24 h	%	10	15	10

* Own study based on the EN 312 standard.

**Table 4:** Results of the TS (24 h) of the particleboards produced.

Unit Pressure, N·mm^−2^	UF Resin Content of the Surface Layers and Core Layer (SL_CL), %	TS, %		
		The Share of Residue Walnut Wood Particles, %		
		0	25	50
1.5	10_8	25.9 (1.32) bc	24.7 (1.08) b	27.2 (1.25) c
2.5		24.3 (0.96) b	24.8 (0.91) b	26.7 (1.21) c
1.5	12_10	21.6 (1.44) a	21.3 (1.42) a	21.4 (1.25) a
2.5		21.7 (1.18) a	20.7 (1.59) a	21.5 (1.27) a

Different letters denote a significant difference. Means followed by the same letter do not statistically differ from each other (*p* ≤ 0.05) according to Tukey’s post hoc test.

## Data Availability

Not applicable.
